# Supplementary material for: A Prognostic Model to Predict Ruxolitinib Discontinuation and Death in Patients with Myelofibrosis
Source: Cancers (Basel). 2023 Oct 17;15(20):5027. doi: 10.3390/cancers15205027 (PMC10605705; doi:10.3390/cancers15205027)
Supplement: Supplementary file 1 [file cancers-15-05027-s001.zip › cancers-2607503-supplementary.pdf]

Supplemental Figure S1. Patients' disposition

***List of abbreviations: F-UP: follow-up; MF: myelofibrosis; RUX: ruxolitinib; ASCT: autologous stem cells transplant.**

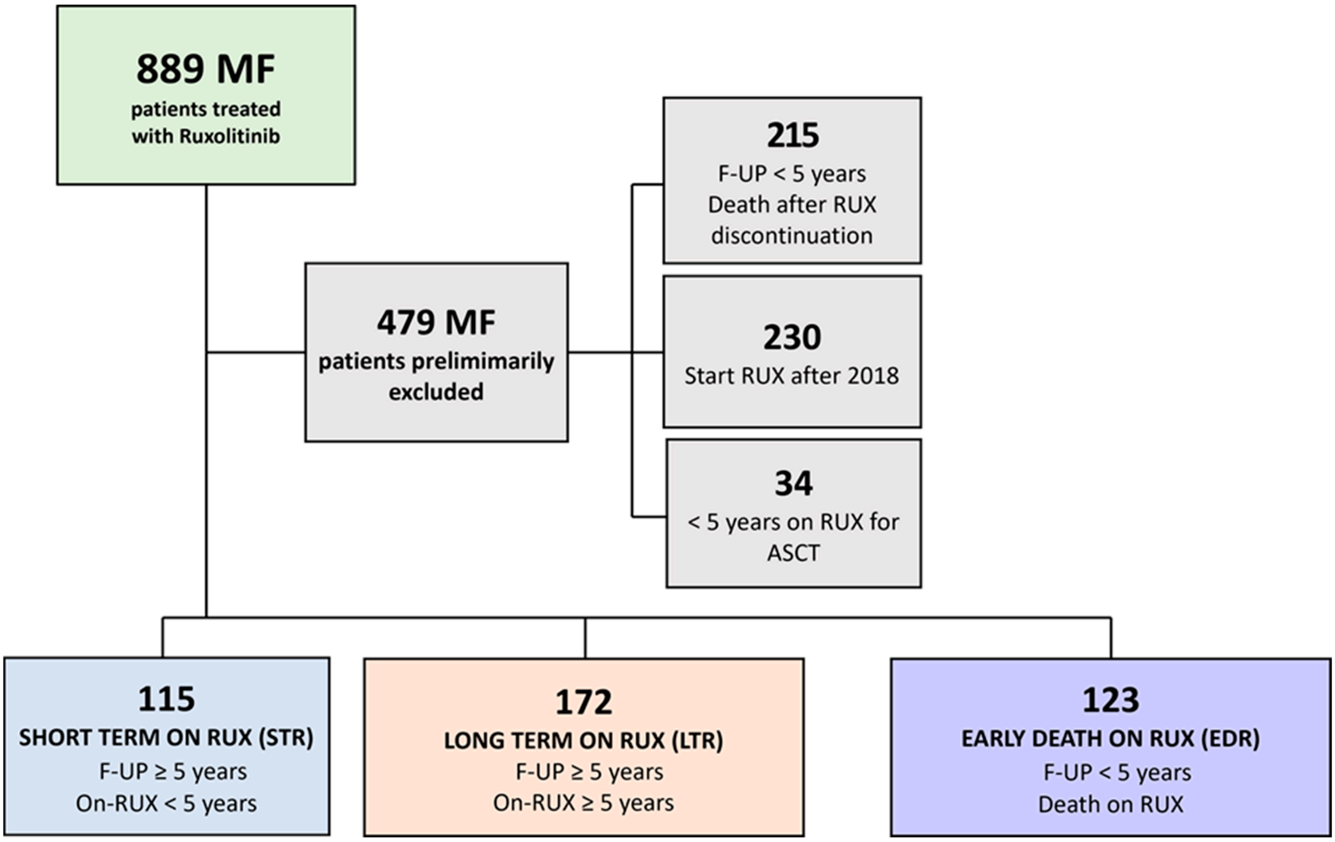

Supplemental Table S1. Univariate and multivariate analysis for discontinuation within 5 years

Variable (at baseline)	Univariate, HR (95% CI); p-value	Multivariate, HR (95% CI); p-value
**Age ≥ 65**	0.84 (0.59-1.22); 0.37	
**Male sex**	1.08 (0.75-1.56); 0.68	
**PLT < 100 x10^9/L**	2.39 (1.28-4.45); **0.006**	2.02 (1.05-3.92); **0.04**
**Hb < 10 g/dL**	1.99 (1.37-2.91); **<0.001**	1.64 (1.07-2.52); **0.02**
**Transfusion dependence**	2.05 (1.32-3.18); **0.001**	1.27 (0.65-2.49); 0.49
**RUX starting dose <10 BID**	2.63 (1.72-4.00); **<0.001**	1.95 (1.22-3.12); **0.005**
**TSS ≥ 20**	1.49 (1.01-2.19); **0.03**	1.28 (0.84-1.97); 0.26
**High/INT-2 DIPSS or MYSEC-PM**	1.24 (0.83-1.85); 0.29	
**Primary MF**	1.59 (1.1-2.30); **0.01**	1.96 (1.28-2.99); **0.002**
**Bone marrow fibrosis ≥ 2**	0.98 (0.63-1.51); 0.93	
**Spleen > 10 cm BLCM**	1.10 (0.76-1.59); 0.62	
**RUX start after DX > 2 yrs**	1.51 (1.05-2.18); **0.03**	1.18 (0.76-1.83); 0.46
**No SR at 3 mos**	**1.57 (1.00-2.50); 0.05**	1.64 (1.02-2.61); **0.04**
